# Rainy and Dry Seasons Are Relevant Factors Affecting Chemical and Antioxidant Properties of Meliponini Honey

**DOI:** 10.3390/foods14020305

**Published:** 2025-01-17

**Authors:** Flavia C. Lavinas, Brendo A. Gomes, Marcos V. T. Silva, Raissa Lima, Suzana G. Leitão, Mirian R. L. Moura, Rosineide C. Simas, Renata F. Barbosa, Fabricio O. Silva, Carla S. Carneiro, Igor A. Rodrigues

**Affiliations:** 1 Programa de Pós-Graduação em Ciências Farmacêuticas, Faculdade de Farmácia, Universidade Federal do Rio de Janeiro, Rio de Janeiro 21941-902, Brazil; flavia.c.lavinas@gmail.com (F.C.L.); mvtoledosilva@gmail.com (M.V.T.S.); raissa08.lima@gmail.com (R.L.); 2 Programa de Pós-Graduação em Biotecnologia Vegetal, Universidade Federal do Rio de Janeiro, Rio de Janeiro 21941-902, Brazil; brendoo.bc@gmail.com; 3 Departamento de Produtos Naturais e Alimentos, Faculdade de Farmácia, Universidade Federal do Rio de Janeiro, Rio de Janeiro 21941-902, Brazil; sgleitao@pharma.ufrj.br (S.G.L.); mirian.rlm@gmail.com (M.R.L.M.); fabricio@pharma.ufrj.br (F.O.S.); 4 Escola de Engenharia, Universidade Presbiteriana Mackenzie, São Paulo 01302-907, Brazil; simas.rc@gmail.com; 5 Departamento Multidisciplinar, Universidade Federal de São Paulo, São Paulo 06120-042, Brazil; renata.barbosa28@unifesp.br

**Keywords:** stingless bee honey, seasonality, honey composition, honey physicochemical properties, antioxidant activity, chemical profile, chemometric analysis

## Abstract

Brazilian stingless bee species produce honey with distinct physicochemical and bioactive properties shaped by environmental factors. This study investigated the effects of the rainy and dry seasons on the physicochemical characteristics, chemical fingerprinting, mineral content, and antioxidant capacity of honey from *Melipona mondury* and *Melipona bicolor*. The honey samples were analyzed for their phytochemical properties (official methods), total phenolics (Folin–Ciocalteu method), flavonoid content (aluminum complex formation method), antioxidant capacity (FRAP and ABTS assays), and antioxidant activity (erythrocyte model). The mineral content was assessed via TXRF spectroscopy, and chemical fingerprinting was conducted using mass spectrometry. Chemometric tools were used for the samples’ discriminating analyses, including Principal Component Analysis (PCA) and Partial Least Squares–Discriminant Analysis (PLS-DA). Seasonal variations significantly affected the moisture, total soluble solids, and acidity. In turn, the antioxidant capacity was influenced mainly by the bee species. The mineral composition, particularly potassium, phosphorus, and calcium, remained stable. Multivariate analysis identified *m*/*z* ions (VIP scores > 2.5), rather than physicochemical or antioxidant capacity parameters, as critical for seasonal discrimination. The antioxidant activity, assessed by oxidative hemolysis prevention, was robust across the seasons, with *M. mondury* honey (2 mg·mL^−1^) from the rainy season outperforming ascorbic acid. These findings underscore the impact of the rainy and dry seasons and the potential of secondary metabolite fingerprinting to identify collection periods.

## 1. Introduction

Brazil harbors a remarkable diversity of stingless bees, with approximately 300 species distributed across its territory. These bees, also known as meliponines or Indigenous bees, are gaining attention for their vital role in maintaining ecosystems and supporting food security, especially in the context of climate change and global warming [1]. Their economic importance is increasingly recognized, particularly in relation to sustainable development and the commercialization of stingless bee products, such as propolis, geopropolis, pollen, and honey. Despite this, stingless beekeeping remains an informal activity, marked by unstandardized and rudimentary management practices, yet it serves as a primary source of income for many Brazilian communities [2].

Among stingless bee products, honey is particularly valued for its high nutritional content and distinctive sensory characteristics, which set it apart from honey produced by other bee species [3]. The uniqueness of stingless bee honey (SBH) stems from the chemical modification of nectar collected from diverse native flora, reflecting the ecological richness of its environment. Furthermore, environmental factors, especially climatic conditions, play a critical role in determining the chemical and physicochemical properties of SBH [4]. However, the impacts of climate change pose substantial challenges to stingless bee populations and their ability to produce honey. Changes in climate disrupt plant–pollinator interactions, reduce food availability, and negatively affect bee survival, metabolism, and gene expression, as recently reviewed [5]. These stressors threaten not only honey production but also the ecological services provided by stingless bees. Tennakoon et al. (2023) provided alarming projections, indicating that climate change could drive significant shifts in bee habitats, with currently highly suitable areas potentially declining to moderate, marginal, or entirely unsuitable conditions by 2020–2039 and 2060–2079 [6]. Such changes could jeopardize the sustainability of SBH production and the livelihoods of communities that depend on it, emphasizing the urgency of addressing climate-related challenges in beekeeping practices.

Several Brazilian research groups have explored the physicochemical, nutritional, and functional features of SBH [7,8,9,10], though only a few studies have specifically examined the impact of seasonal changes. De Sousa et al. (2016) reported that monofloral honey produced by *Melipona subnitida* and *Melipona scutellaris*, collected during the dry and rainy seasons in the Brazilian semi-arid region, exhibited distinct chemical and sensory profiles [3]. This variation is likely due to the bees’ preference for floral species, which are available only during specific seasons. More recently, Da Sant’ana et al. (2020) demonstrated that seasonality was a key factor in distinguishing honey from *M. subnitida* and *Melipona fasciculata* in the same region [11]. It is noteworthy that the diverse composition of SBH poses challenges for standardization, highlighting the need for more comprehensive studies to better understand and regulate these unique honeys.

SBH has been documented as beneficial to human health owing to its elevated antioxidant content [12]. The advantageous properties of honey are ascribed to its array of antioxidant compounds, encompassing amino acids, proteins, enzymes, carotenoids, organic acids, and polyphenols, notably flavonoids and phenolic acids [2,13]. The composition of SBH has garnered significant attention from researchers due to its potential in averting ailments linked to oxidative stress. Furthermore, it exhibits therapeutic properties, including wound healing, antiseptic, anticancer, and anti-inflammatory effects [4].

Seasonality can influence the properties of stingless bee honey, affecting its antioxidant, antimicrobial, and physicochemical characteristics [14,15]. Understanding how seasonality influences the properties of stingless bee honey can help with the planning of collection periods and offer a more standardized product. This knowledge is also essential to support the creation of specific regulatory standards for stingless bee honey [16]. These standards are essential to prevent adulteration, ensure the product’s authenticity, and promote its recognition in national and international markets.

Recognizing that comparative studies on honey involve various techniques, rather than solely relying on univariate analyses of samples, precise and innovative mathematical and statistical approaches have become increasingly important [17]. Data analyses such as chemometric evaluations can be effectively applied in SBH characterization. This study investigated, through a chemometric approach, the influence of seasonality (the rainy and dry seasons) on the physicochemical characteristics, mineral profile, and antioxidant properties of honey from *Melipona mondury* and *Melipona bicolor*.

## 2. Materials and Methods

### 2.1. Chemicals

Folin–Ciocalteu reagent, gallic acid, quercetin, TPTZ (2,4,6-tripyridyl-s-triazine), ABTS [(2,20-azinobis-(3-ethylbenzothiazoline-6-sulfonic acid), AAPH (2,2′-Azobis(2-methylpropionamidine) dihydrochloride], and nitric acid were purchased from Sigma-Aldrich Co (St. Louis, MO, USA). High-performance liquid chromatography (HPLC)-grade chloroform and acetonitrile were obtained from Tedia Brazil^®^ (Rio de Janeiro, RJ, Brazil). All the other chemicals were analytical-grade.

### 2.2. Stingless Bee Honey (SBH) Samples

Honey samples from *M. mondury* and *M. bicolor* were provided by a meliponary located at Guapimirim (RJ, Brazil). The samples were collected in distinct periods: December to March, corresponding to the rainy season (summer and spring), and April to July, corresponding to the dry season (autumn and winter), between 2018 and 2021. This seasonal classification was based on climatological data for the region, including the average temperature (°C), rainfall (mm), humidity (%), and number of rainy days (https://pt.climate-data.org/, accessed on 22 January 2022). Only mature honey from sealed honey pots was collected, ensuring that the honey had reached full ripeness and stability. To further strengthen the seasonal analysis, the collection schedule respected a minimum interval of one month after the start of each season, ensuring that the bees had consumed honey produced during the previous season. This strategy allowed for the collection of honey representative of the current season’s environmental and floral conditions. All samples were collected directly from the closed honey pots and stored in darkness at 4 °C until the analytical procedures were performed. Table 1 summarizes the samples’ information.

### 2.3. Physicochemical Analyses

The physicochemical properties of the SBH, including the moisture, water activity (Aw), total soluble solids (TSS), pH, total and free acidity, and 5-hydroxymethylfurfural (HMF), were determined following the procedures established by the Association of Official Analytical Chemists [18]. The SBH color was determined spectrophotometrically (0.5 g of honey in 1 mL of distilled water) at 635 nm, and the results were converted to the Pfund scale (mm) as previously described [19]. In addition, the presence of dark pigments was determined by measuring the absorbance at 420 nm of honey samples previously diluted to 4 °Brix [20].

### 2.4. Multi-Element Analysis of Minerals

The content of minerals and trace elements in the SBH was determined using Total Reflection X-Ray Fluorescence (TXRF). Honey solutions were prepared by adding 1% Triton and 20 μL of the internal standard gallium to 1.0 g of honey sample. The internal standard is necessary to quantify elements by TXRF [21]. The samples were then homogenized using a vortex mixer. An aliquot of 10 μL from each solution was pipetted onto the center of a quartz reflector support and then dried in an oven at 60 °C. The samples were prepared and measured in triplicates. The blank samples were prepared the same way as the honey samples. All measurements were conducted using the S2 Picofox spectrometer (Bruker^®^, Berlin, Germany) The measurement time was 300 s, the voltage was 40 kV, and the X-ray tube was 50 W, and a molybdenum target and silicon drift detector were used.

### 2.5. Phenolic Determination

The total phenolic (TPC) and flavonoid (TFC) contents were determined spectrophotometrically with the Folin–Ciocalteu [22] and flavonoid–aluminum complex formation [19] methods, respectively. Honey sample solutions (1:10 in distilled water, *w*/*v*) were prepared and filtered through qualitative Whatman^®^ (Maidstone, UK) Grade 1 filter paper (11 μm). Aliquots of 0.1 mL were taken from the samples or previously prepared gallic acid solutions (standard, 7 to 200 μg·mL^−1^) and mixed with 0.5 mL of Folin–Ciocalteu reagent (10% *w*/*v*) and 0.4 mL of sodium bicarbonate (7.5% *w*/*v*). After 2 h of incubation in the dark at room temperature, absorbances were measured at 760 nm (SpectraMax M2, Molecular Devices, Sunnyvale, CA, USA). TPC results were expressed in milligrams of gallic acid equivalents per 100 g of honey in fresh weight (mg GAE·100 g^−1^). The TFC content was determined from a honey solution (0.25 g in 1.25 mL of distilled water and 75 μL of 5% NaNO_2_) mixed with 0.15 mL of a 10% AlCl_3_ solution. After 5 min, 0.5 mL of 1 M NaOH solution was added, and the final volume was fit to 2.5 mL with distilled water. The absorbance of the sample was measured at 510 nm using a SpectraMax M2 microplate reader (Molecular Devices, Sunnyvale, CA, USA). Quercetin was used as a standard (50–550 μg·mL^−1^), and the TFC was expressed as milligrams of quercetin equivalents per 100 g of honey in fresh weight (mg QE·100 g^−1^).

### 2.6. Antioxidant Capacity Analysis

The SBH antioxidant capacity was evaluated using the FRAP (ferric-reducing antioxidant power) method [23] with some modifications. In brief, 20 μL of honey solution (100 mg·mL^−1^) was added to 180 μL of freshly prepared FRAP reagent (2 mL of 10 mM TPTZ solution in 6N HCl, 2 mL of 20 mM FeCl_3_ solution, and 20 mL of 300 mM acetate buffer, pH 3.6, preheated at 37 °C). The reaction mixture was then incubated at 37 °C for 4 min before measuring the absorbance at 595 nm. A ferrous sulfate solution was used as a standard (50–1000 μM), and FRAP values were expressed in micromoles of ferrous per 100 g of honey in fresh weight (μmol Fe^2+^·100 g^−1^).

For the radical scavenging assay, 10 μL from each diluted honey sample was mixed with 190 μL of ABTS^●+^ solution, which was prepared as previously described [24]. The assay was conducted in a microplate, with 10 μL of ABTS reagent and 10 μL of ultrapure water (blank), as well as the addition of standards or samples, followed by 190 μL of the ABTS radical solution. Absorbance measurements were then taken at 720 nm using a SpectraMax M2 microplate reader. A calibration curve was prepared using Trolox as a standard (0.004 to 0.25 μmol Trolox·L^−1^), and the results were expressed in mM Trolox equivalent antioxidant capacity per 100 g of honey in fresh weight (mmol TEAC·100 g^−1^).

### 2.7. Hemolytic Activity

The hemolytic activity of the SBH was assessed prior to the antioxidant activity assays to ensure their safe usage. Fresh sheep (*Ovis aries*) blood samples collected in K_2_EDTA BD Vacutainer^®^ tubes were purchased from EBE Pharma Biológica e Agropecuária (Rio de Janeiro, RJ, Brazil). The blood was washed four times by centrifugation (2000 rpm, 5 min) with cold phosphate-buffered saline (PBS) (pH 7.4). The erythrocyte pellets were resuspended to a 2% (*v*/*v*) concentration in cold PBS. Subsequently, 80 µL of the suspension was added to each well of a 96-well microplate preloaded with 20 µL of SBH solution (2 mg·mL^−1^, final concentration). The cells were incubated at 37 °C for 4 h with gentle agitation every hour. After incubation, 200 µL of PBS was added to each well to prevent further hemolytic activity. Untreated cells diluted with PBS or distilled water were used as negative and positive hemolysis controls, respectively. The microplate was centrifuged (2000 rpm, 5 min), and the supernatants (200 µL) were transferred to new microplates for the spectrophotometric analysis of the hemoglobin release at 570 nm using a Multiscan FC microplate reader (Thermo Fisher Scientific, Waltham, MA, USA). The animal study protocol was approved by the Institutional Ethics Committee of the Universidade Federal do Rio de Janeiro (CEUA-UFRJ 122/24).

### 2.8. Antioxidant Activity

The antioxidant activity of the SBH was assessed by its protective effect against hemolysis induced by oxidative stress using AAPH, a reactive oxygen species generator [25]. Sheep blood samples were obtained as described above. An erythrocyte suspension (10%, *v*/*v*) in PBS was prepared, and 1 mL aliquots were transferred to Erlenmeyer flasks containing 4 mL of different concentrations of honey (0.12 to 2 mg·mL^−1^) and 7.4 mM AAPH. The AAPH concentration represents the half-maximal oxidative concentration (OC_50_), where 50% of erythrocytes lyse after 4 h of exposure to AAPH at 37 °C. Ascorbic acid (1 µg·mL^−1^) was used as a reference antioxidant. The cells were incubated in a Dubnoff shaking water bath (QUIMIS^®^, São Paulo, Brazil) for 4 h at 37 °C. After incubation, 500 µL of each sample was diluted with cold PBS (1:3) to halt AAPH decomposition into free radicals. Untreated cell suspensions were diluted in cold PBS or distilled water to serve as negative and positive hemolysis controls, respectively. After centrifugation, the supernatants (200 µL) were transferred to 96-well microplates and oxidative stress-induced hemolysis was quantified by the spectrophotometric analysis of the hemoglobin release at 570 nm (Multiscan FC, Thermo Fisher Scientific, Waltham, MA, USA). The results were expressed as the percentage of oxidative stress-induced hemolysis relative to the positive control.

### 2.9. Direct Injection Mass Spectrometry (DI-MS) Analysis

The honey samples (1 g·mL^−1^ in ultrapure water) underwent liquid–liquid partitioning with chloroform (1:1, *v*/*v*). The organic fraction was collected, dried, and stored at −20 °C following phase separation until spectrometric analysis. For this purpose, the samples were dissolved in acetonitrile (2 mg·mL^−1^), and approximately 2 μL was directly injected into an LCQ Fleet mass spectrometer (Thermo Fisher Scientific) equipped with an electrospray ionization (ESI) source operating in positive-ionization mode. Automated direct injection was performed at a flow rate of 0.1 mL·min^−1^ for 5 min. High-purity nitrogen was used as sheath gas and auxiliary gas, while high-purity helium was used as collision gas. The DI-MS parameters consisted of a source voltage of 5 kV, a source current of 100 μA, a source temperature of 450 °C, a capillary voltage of 7 V, a tube lens voltage of 65 V, and a capillary temperature of 400 °C. MS spectra were acquired in the range of *m*/*z* 50–1000.

### 2.10. Statistical Analyses

The honey samples (1 g·mL^−1^ in ultrapure water) underwent liquid–liquid partitioning with chloroform (1:1, *v*/*v*). The organic fraction was collected, dried, and stored at −20 °C following phase separation until spectrometric analysis. Analyses of the physicochemical properties, mineral and total bioactive contents, and antioxidant capacity of the honey samples were performed in triplicate three times independently, and the results were expressed as mean values ± standard deviation (SD). The antioxidant activity assay was performed in duplicate, three times independently, and the results were expressed as mean values ± standard error (SE). The Shapiro–Wilk test was conducted to assess the normality of the data (*p* ≥ 0.05 indicating normal distribution). For data that followed a normal distribution (parametric data), one-way ANOVA was applied, followed by Tukey’s post hoc test for pairwise comparisons. Non-parametric data were analyzed using the Kruskal–Wallis and Dunn’s post hoc tests for multiple comparisons. Statistical significance was set at *p* ≤ 0.05. Correlations between variables were evaluated using the Spearman correlation coefficient, with a *p*-value ≤ 0.05 considered statistically significant. Statistical analyses were performed using XLSTAT^®^ (version 2014, Addinsoft, Paris, France) or BioEstat 5.0^®^ software.

A chemometric approach was adopted to distinguish honey samples collected from different stingless bee species across rainy and dry seasons. Initially, each sample’s mass spectrometry data (DI-MS) were pre-processed using XCalibur^®^ 2.2 software (ThermoScientific, Waltham, MA, USA). At this stage, *m*/*z* values were adjusted for unit resolution parameters, and the one thousand most intense peaks were exported in centroid mode. To mitigate potential biases, independent variables (*m*/*z* ions) absent in more than one sample per species were discarded. The resulting data (206 ions) were combined with three other datasets, consisting of ten variables representing physicochemical parameters, eight related to concentrations of the analyzed minerals, and four representing antioxidant activity data. Thus, the working matrix was formed with 228 factors. The data were scaled, normalized, and mean-centered for the joint evaluation. Subsequently, multivariate (PLS-DA) and univariate (one-way ANOVA and Tukey HSD post hoc test) analyses were conducted using the MetaboAnalyst 6.0 webserver (https://www.metaboanalyst.ca/, accessed on 18 April 2023). For this purpose, the samples were dissolved in acetonitrile (2 mg·mL^−1^), and approximately 2 μL was directly injected into an LCQ Fleet mass spectrometer (Thermo Fisher Scientific) equipped with an electrospray ionization (ESI) source operating in positive-ionization mode. Automated direct injection was performed at a flow rate of 0.1 mL·min^−1^ for 5 min. High-purity nitrogen was used as sheath gas and auxiliary gas, while high-purity helium was used as collision gas. The DI-MS parameters consisted of a source voltage of 5 kV, a source current of 100 μA, a source temperature of 450 °C, a capillary voltage of 7 V, a tube lens voltage of 65 V, and a capillary temperature of 400 °C. MS spectra were acquired in the range of *m*/*z* 50–1000.

## 3. Results and Discussion

### 3.1. Physicochemical Properties of SBH

The physicochemical parameters of the SBH produced by the species *M. mondury* and *M. bicolor* in relation to the dry and rainy seasons are presented in Table 2. When analyzing the same bee species across different seasons, no significant difference in the moisture was observed (*p* ≥ 0.05). However, when comparing different species within the same season (dry or rainy), *M. bicolor* showed a higher water content (31.56%) compared to that of *M. mondury* during the dry season (27.25%) (*p* ≤ 0.05). The moisture levels in the honey of the studied species ranged from 32.74% to 35.76% during the rainy season and from 27.25% to 31.56% during the dry season. A similar pattern was observed for the water activity (Aw), another important parameter related to the water content in honey, with values ranging from 0.79 to 0.80 during the rainy season and from 0.72 to 0.75 in the dry season. In addition, the value determined for the Aw of *M. mondury* honey was significantly higher (Aw = 0.8) during the rainy season compared to that of the dry season (Aw = 0.72) (*p* ≤ 0.05). Variations in the water content of SBH may be influenced by the characteristics of the biome where the bees are found. The high pluviometric index during the rainy season can affect the water content in plant nectars, which, in turn, may impact the honey produced. Additionally, high relative humidity contributes to increased moisture and water activity (Aw) in honey due to its hygroscopic characteristics [26]. Marcolin et al. (2021) showed higher moisture values for honey collected in the rainy season compared to those for honey collected in the dry season [27].

When comparing the regulated physicochemical parameters for *A. mellifera* honey with those reported for stingless bee honey (SBH), the moisture content emerges as the most significant difference. The Codex Alimentarius and Brazilian legislation sets a maximum moisture limit of 20% for *A. mellifera* honey [28,29], whereas the moisture levels reported in the scientific literature for SBH are higher [9,11,30,31]. This discrepancy highlights the need for specific regulations for SBH. According to Nascimento et al. (2015), a high moisture content is a characteristic factor of SBHs, especially for those produced by species belonging to the genus *Melipona* [10]. Furthermore, the high percentage of humidity may be due to the honey operculation process, which is performed by these bees at a higher humidity than *A. mellifera* bees [32].

For the total soluble solids (TSS), the levels were significantly higher during the dry season compared to the rainy season for the honey of *M. mondury* (*p* ≤ 0.05). In contrast, the *M. bicolor* honey showed no significant difference between the collection periods (*p* ≥ 0.05). Considering the two species studied, the average values ranged from 61.55 to 70.99 °Brix for *M. mondury* and from 64.88 °Brix to 69.43 °Brix for *M. bicolor*. Significant differences were observed only in the dry season when comparing the different species in the same season (*p* ≤ 0.05). In honey and other sugary foods, a lower moisture content typically correlates with higher TSS levels [33]. This pattern was observed in the present study, where the honey with the lowest moisture content (27.25%), the *M. mondury* honey collected in the dry season, presented the highest TSS content, with 70.9 °Brix. Similar TSS results to those observed in the present study were reported for stingless bee honey from southern Brazil, with values ranging from 69.1 to 69.6 °Brix for *M. mondury* and from 60.1 to 76.1 °Brix for *M. bicolor* [9].

In the present study, it was observed that the total acidity content was influenced by the seasons. When evaluating the species studied, the average total acidity values ranged from 89.18 to 247.35 mEq·Kg^−1^ for *M. mondury* honey and from 195.20 to 270.69 mEq·Kg^−1^ for *M. bicolor* honey during the dry and rainy seasons, respectively. Significant differences were observed for the same species in different seasons (*p* ≤ 0.05) (Table 2). Regarding the free acidity, only the honey produced by *M. mondury* showed significant differences depending on the seasonality (*p* ≤ 0.05). In this honey, the mean free acidity in the dry period was 46.01 mEq·Kg^−1^, while in the rainy period, it was 91.57 mEq·Kg^−1^ (Table 2). There was also a significant difference in the free acidity contents during the dry season between the species, with values of 46.01 mEq·Kg^−1^ for *M. mondury* honey and 106.73 mEq·Kg^−1^ for *M. bicolor* (*p* ≤ 0.05). These results may indicate that specific factors related to each species influence this parameter. In this context, it is known that honey acidity is a variable parameter that can be influenced by the balance of organic acids, geographical origins, floral species, and bee species [3]. It is also worth noting that high acidity levels may indicate that the honey is undergoing fermentation, potentially affecting its quality and sensory attributes [13]. However, fermentation is a natural characteristic of SBH and may even be seen as a positive factor, as it can increase the bioactivity of the honey [34,35]. Relevant variations in the acidity values of SBH are commonly recorded in the literature. Nonetheless, the free and total acidity levels described here align with those cited for other Brazilian SBH [9,27,30,34,35].

In the present study, when comparing the pH values for honey of different species within the same collection period, these varied from 3.15 to 3.36 in the rainy season and remained at 3.50 in the dry season. No significant difference existed between the species in the same season (*p* ≥ 0.05). The mean pH value was significantly higher in the dry season compared to that of the rainy season for the species *M. mondury* (Table 2) (*p* ≤ 0.05). There was no significant difference for the species *M. bicolor* (*p* ≥ 0.05). A study on SBH from Malaysia reported no significant impact of the rainy and dry seasons on the pH levels [36]. The pH variation of honey is also often influenced by its geographic origins and botanical and mineral composition [34]. It is highly influenced by the pH of soil and nectar [37]. Notably, pH values similar to those obtained in the present study were reported by other research groups when analyzing SBH [11,38,39].

The average dark pigments and HMF values for honey collected during the rainy and dry seasons did not show significant differences (*p* ≥ 0.05). The values of the dark pigments ranged from 0.13 to 0.14 for the rainy season and from 0.13 to 0.17 for the dry period. The HMF values ranged from 4.26 to 5.35 mg·Kg^−1^ in honey samples collected during the rainy period, while those gathered in the dry season displayed a range from 1.26 to 1.92 mg·Kg^−1^ (Table 2). Similar results for the levels of dark pigments obtained in this study were reported when analyzing SBH from the southern region of Brazil [40]. Regarding HMF, both the values found in the present study and those reported previously for SBH are low when compared to the maximum limit established by the *Codex Alimentarius* for *A. mellifera* honey (40 mg·Kg^−1^) [7,28,41]. Biluca et al. (2014) suggest a possible resistance of SBH to HMF formation, which could prolong the product’s shelf life [42]. In the present study, these parameters had no significant differences when comparing honey from different species within the same season (*p* ≥ 0.05). The non-influence of the seasonality and species on the HMF levels may be associated with other variables that were not evaluated in this study, including the storage conditions, such as the heating of the honey, prolonged storage time, low pH, and use of metal containers [31,43]. It is also important to highlight that the honey used in this study was collected fresh and promptly stored at low temperatures.

Regarding the color parameter, the dry or rainy collection period significantly influenced only the honey produced by *M. bicolor*, with average values of 8.32 mm for the rainy season and 37.34 mm for the dry period using the Pfund scale. In this study, the color profile ranged from extra light amber to extra white, potentially reflecting the collection region’s floral diversity and mineral content. Indeed, the color of honey is predominantly influenced by its botanical origin, mineral composition, and storage time [44].

### 3.2. Mineral Profile

The results obtained for the mineral profiles in the honey samples of *M. mondury* and *M. bicolor* collected during the rainy and dry seasons are presented in Table 3. When analyzing each species separately, no statistical difference was observed between the mineral levels in the two evaluated collection seasons (*p* > 0.05). However, when comparing the honey produced by different species of SBs, a significant difference was observed between the samples for most elements (P, K, and Ca) (*p* < 0.05). This result may be related to differences in the bees’ foraging behavior, physiology, or preferred floral sources. Previous studies suggest that bees of different species forage on different nectar sources, which can lead to variations in the mineral content of honey, as the mineral composition of nectar depends on the plant’s mineral absorption [9,45]. In fact, our group demonstrated that the iron content of Brazilian stingless bee honey (SBH) varied significantly according to the biogeographical zone in which it was collected [7]. In addition, bees can act as potential environmental indicators, as they travel long distances and carry contaminants that influence the mineral content of the honey they produce [46].

Regarding elements that may pose health risks, such as Al, Cd, Pb, Hg, and Ni, none of the analyzed SBH samples contained these contaminants. These elements can originate from natural or anthropogenic sources, such as environmental pollution and industrial activities [47]. However, the samples contained Rubidium (Rb), a non-essential element rarely investigated in the food chain. In nature, its abundance is comparable to that of zinc, as it is widely distributed in the Earth’s crust, where it can be found in minerals along with potassium. Geological origins significantly influence the Rb content of plants and may consequently affect the Rb content of honey [48,49]. Batista et al. (2012) reported in their study on Brazilian honey that the Rb content is associated with the floral origin, agricultural practices, and soil characteristics [50].

From a nutritional point of view, the consumption of SBH has aroused interest due to its benefits, including its mineral content [49]. The most abundant chemical element in the honey samples in this research was potassium (K), with values ranging from 48.18 to 266.15 mg·Kg^−1^. Potassium is essential for a healthy nervous system and regular heart rhythm, reducing blood pressure and the risk of cardiovascular disease, stroke, and coronary artery disease in adult humans [50]. The other two minerals that stood out quantitatively in the samples were P and Ca, with average values ranging from 13 to 94.16 mg·Kg^−1^ and from 15.58 to 49.08 mg·Kg^−1^, respectively, which are also considered important for human nutrition. The mineral content was as follows in descending order: K > P > Ca > Fe > Mn > Cu > Zn.

In this study, the mineral intake from stingless bee honey (SBH) was estimated based on the Dietary Reference Intakes (DRIs) for minerals, as outlined by the U.S. National Institute of Health [51]. Honey consumption recommendations were considered according to the American Heart Association (AHA) guidelines on limiting added sugar intake, which includes honey [52], and the Brazilian Health Regulatory Agency (ANVISA) [53]. The AHA recommends 37 g of added sugar per day for men and 25 g for women, while the ANVISA suggests a portion of 20 g for adults. Based on the Brazilian portion size, the analysis showed that the mean daily intake of the evaluated minerals would be 3.01 mg of potassium (K), 0.91 mg of phosphorus (P), 0.51 mg of calcium (Ca), 0.07 mg of iron (Fe), 0.02 mg of manganese (Mn), 0.0156 mg of copper (Cu), and 0.007 mg of zinc (Zn). These values are comparable to those estimated using the AHA recommendations, particularly for women.

**Table 3:** Mineral profile of *M. mondury* and *M. bicolor* honey collected during the rainy and dry seasons in the Atlantic Forest region.

SBH	Season	Minerals (mg·Kg^−1^)							
		P	K	Ca	Mn	Fe	Cu	Zn	Rb
*M. mondury*	Rainy	13.00 ^A,a^ (7.44–19.72)	105.57 ^A,a^ (49.79–158.99)	15.58 ^A,a^ (19.70–20.09)	0.69 ^A,a^ (0.05–1.54)	0.85 ^A,a^ (0.12–1.75)	nd	nd	2.25 ^A,a^ (0.82–3.68)
	Dry	94.16 ^A,a^ (10.52–200.7)	48.18 ^A,a^ (21.87–75.40)	18.77 ^A,a^ (3.33–36.46)	0.57 ^A,a^ (0.16–0.99)	3.88 ^A,a^ (1.06–6.77)	0.11 (nd–0.23)	0.17 (nd–0.35)	0.64 ^A,a^ (0.52–0.77)
*M. bicolor*	Rainy	52.58 ^A,b^ (24.67–80.59)	266.15 ^A,a^ (144.93–392.90)	49.08 ^A,b^ (23.92–74.63)	3.31 ^A,a^ (1.21–5.64)	5.29 ^A,b^ (2.97–7.65)	2.07 ^A,a^ (nd–4.23)	0.85 ^A,a^ (nd–1.83)	2.86 ^A,a^ (nd–4.41)
	Dry	21.34 ^A,a^ (17.00–80.58)	182.38 ^A,b^ (102.28–260.98)	18.31 ^A,a^ (15.32–21.53)	1.14 ^A,a^ (1.02–1.28)	4.39 ^A,a^ (0.71–8.09)	0.17 ^A,a^ (nd–0.35)	0.02 ^A,a^ (nd–0.04)	1.96 ^A,a^ (1.69–2.41)
Average mineral content ^¥^		45.27	150.57	25.44	1.43	3.60	0.78	0.35	1.93
Mineral consumption relative to DRI *	Men (37 g **)	0.24	0.17	0.09	2.22	1.66	3.14	0.11	---
	Women (25 g **)	0.16	0.15	0.05	2.00	1.12	2.12	0.11	---

Values are expressed as mean, minimum, and maximum values. To check the differences between the means, the Kruskal–Wallis test was performed, followed by Dunn’s post hoc test (*p* < 0.05). ^A,B^ Different capital letters superscripted in the same column show significant differences between the same species in different seasons (i.e., compare the same species in different seasons). ^a,b^ Different superscript lowercase letters in the same column show significant differences between the different species in the same seasons (i.e., compare different species in the same season). ^¥^ The average mineral content represents the mean value of each mineral, accounting for variations in producing bee species and seasons. This value was calculated to estimate the percentage of mineral consumption relative to Dietary Reference Intakes (DRIs). * Percentage of mineral consumption relative to DRI values for minerals, as defined by the U.S. National Institute of Health [51]. ** American Heart Association (AHA) recommendation for added sugar intake, including honey, expressed in grams: 37 g for men and 27 g for women [52]. nd, not detected. --- There is no established reference value.

Although K, P, and Ca were the main elements in the SBH, their percentage contribution to human nutrition is modest, providing less than 1% of the DRI, regardless of the health agency recommendation used for the calculation. As shown in Table 3, Fe contributed to the DRI by 1.12% to 1.66%, while Zn’s contribution was 0.11%. Additionally, the consumption of SBH could provide 2.22% and 2.00% of the DRI for Mn and 3.14% and 2.12% for Cu in men and women, respectively. It is worth noting that honey, rich in sugars, is often consumed alongside other foods, promoting a more balanced mineral intake [54]. However, it is important to acknowledge that the daily consumption values presented in this study are theoretical, as the actual absorption depends on mineral interactions within the digestive system. Therefore, further research on the bioaccessibility and bioavailability is needed.

### 3.3. Total Phenolic Compounds, Total Flavonoids, and Antioxidant Capacity of SBH

**Figure 1:**
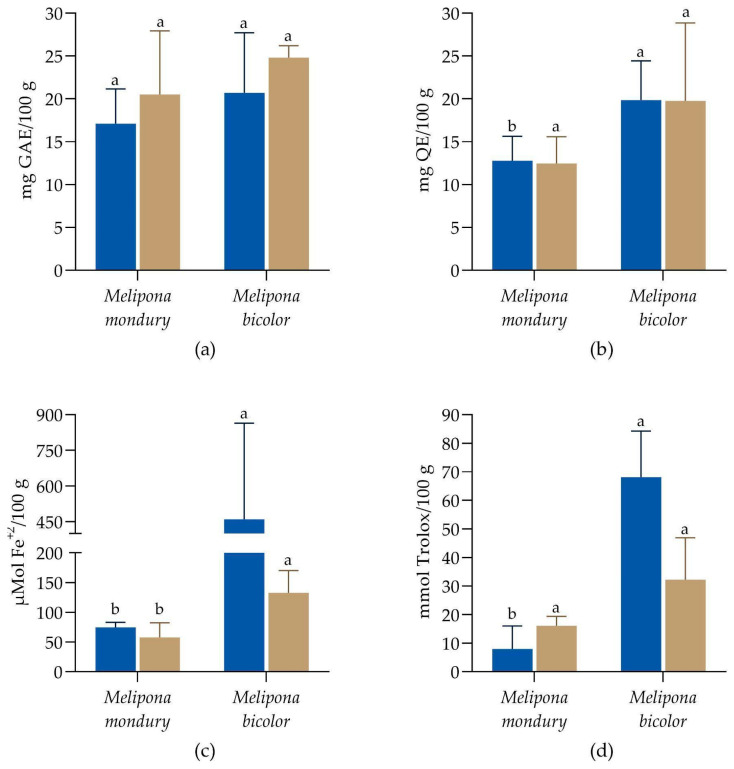
Total phenolic compounds (**a**), total flavonoids (**b**), FRAP values (**c**), and ABTS values (**d**) of honey samples from *M. mondury* and *M. bicolor* obtained from rainy (■) and dry (■) seasons. Differences between samples were considered significant when *p* < 0.05 (Kruskal–Wallis and Dunn post hoc tests). There was no statistical difference when the dry and rainy seasons were compared. Superscript letters represent differences between samples from different species and the same season.

The values of the TPC (mg GAE·100 g^−1^), TFC (mg QE·100 g^−1^), and antioxidant capacity are presented in Figure 1. The TPC values ranged from 17.1 to 24.8 mg GAE·100 g^−1^ in the samples. Considering the different seasons, rainy or dry, and the honey type produced by *M. mondury* or *M. bicolor*, there were no statistical differences among the samples for the TPC (Kruskal–Wallis and Dunn’s post hoc tests, *p* < 0.05). For the TFC values, there was a statistical difference between the honey types in the rainy season, and the remaining values were statistically equal (*p* < 0.05). The *M. mondury* honey from the rainy season showed 12.8 mg QE·100 g^−1^ of the sample, while the *M. bicolor* honey obtained from the same season showed higher values (19.8 mg QE·100 g^−1^). For the antioxidant capacity by FRAP assay, no statistical significance was observed among the honey samples collected from the different seasons, regardless of the bee species (*p* > 0.05). Despite this, there was a significant difference between the samples, with higher FRAP values determined for the *M. bicolor* honey, ranging from 57.9 to 460.4 μmol Fe^+2^·100 g^−1^, and ABTS values ranging from 7.9 to 68.1 mmol Trolox·100 g^−1^.

Stingless bee honey is known for its composition of bioactive molecules, especially the phenolic compounds, which play a crucial role in the antioxidant potential of honey [34]. Agussalim et al. (2022) showed that honey from *Tetragonula laeviceps*, a stingless bee native to Asia, obtained from different geographical origins, presented TPC values ranging from 0.65 to 2.30 mg GAE·100 g^−1^ and TFC values ranging from 0.25 to 1.0 mg QE·100 g^−1^ [55]. The honey samples from the present study have much higher values, suggesting that Brazilian native bee honey is a good source of bioactive constituents such as phenolic compounds. The phenolic composition of honey can vary widely depending on factors such as the floral source, geographical location, climate, and processing methods [34]. Different floral nectars contain distinct phenolic profiles, leading to variations in the types and concentrations of phenolic compounds present in honey. Also, the phytochemical composition of honey can be influenced by the presence of phytochemicals in the wax. Wax is a product of the plant resin that stingless bees collect to build the pots where the honey will be produced [56]. Despite this, our results suggest that the season did not affect the composition of phenolic compounds or antioxidant capacity of the honey.

### 3.4. Correlation Analysis

Table S1 shows the correlation matrix. A significant correlation was observed between some physicochemical and antioxidant parameters. The moisture content (−0.95) and water activity (Aw) (−0.90) showed a strong inverse correlation with the TSS content (*p* ≤ 0.05). In general, lower TSS values are linked to higher moisture levels, which may contribute to preventing crystallization. Our research group reported similar findings when analyzing stingless bee honey from different biogeographical zones of Brazil [7]. In addition, the free acidity showed a significant correlation with the moisture content (0.86) and water activity (Aw) (0.80) (*p* ≤ 0.05). The correlation between the moisture content and free acidity can be explained in part by the fact that increased moisture in honey promotes microbial growth, triggering the fermentation process.

A significant correlation was also found between the color and total flavonoids (0.48) (*p* ≤ 0.05), suggesting that these components may influence the color. Moniruzzaman et al. (2013) reported that the intensity of the honey color is a consistent parameter indicating the presence of pigments with antioxidant activities, including flavonoids [57]. Indeed, the TPC showed a significant correlation with the FRAP (0.52), while the TFC correlated with both the FRAP and ABTS (0.50 and 0.64, respectively) (*p* ≤ 0.05), indicating that flavonoids are important to honey’s antioxidant activity. The ABTS activity also had a strong correlation with the FRAP (0.77). Padmanabhan and Doss (2023) observed a strong correlation between the antioxidant activity and flavonoid content in their study on SBH, emphasizing the role of flavonoids in antioxidant mechanisms [58].

### 3.5. Antioxidant Activity

**Figure 2:**
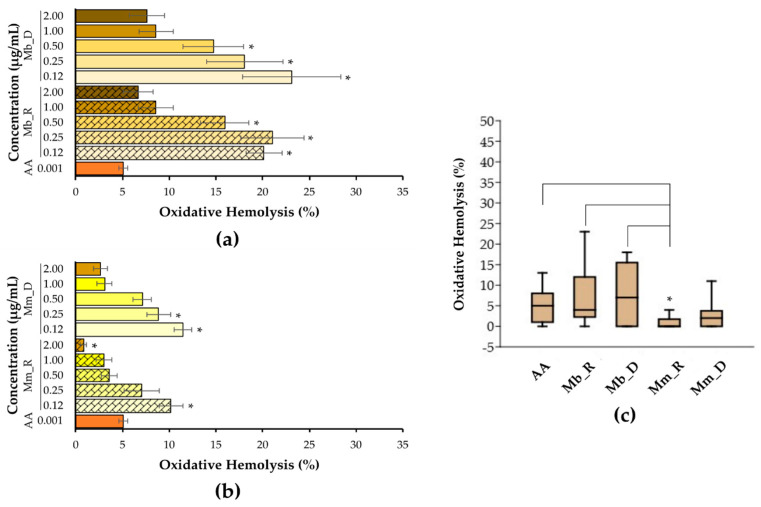
Antioxidant activity of *M. bicolor* and *M. mondury* honeys collected during the rainy and dry seasons. Oxidative hemolysis was chemically induced using 7.0 mM AAPH (OC_50_) for 4 h at 37 °C. (**a**) Oxidative hemolysis prevention by *M. bicolor* honey from the rainy season (hatched colors) and dry season (solid colors); (**b**) oxidative hemolysis prevention by *M. mondury* honey from the rainy season (hatched colors) and dry season (solid colors); and (**c**) comparison between the highest antioxidant concentrations (2 mg·mL^−1^) of honey samples from different seasons and AA (reference antioxidant, 0.001 mg·mL^−1^). Asterisks in (**a**,**b**) indicate a significant difference (*p* < 0.05, Kruskal–Wallis and Dunn tests) between the honey and AA in preventing oxidative hemolysis. The asterisk in (**c**) indicates superior antioxidant activity (*p* < 0.05, Kruskal–Wallis and Dunn tests) of the Mm_R sample compared to that of the Mb_R, Mb_D, and AA. Mb_R: *M. bicolor* honey collected in the rainy season; Mb_D: *M. bicolor* honey collected in the dry season; Mm_R: *M. mondury* honey collected in the rainy season; Mm_D: *M. mondury* honey collected in the dry season; AA: ascorbic acid.

The antioxidant activity of the *M. bicolor* and *M. mondury* honeys was evaluated based on their ability to prevent oxidative hemolysis induced by AAPH at OC_50_ (7 mM). As shown in Figure 2, both honeys inhibited oxidative hemolysis across all the tested concentrations, regardless of the season of collection (rainy or dry). These results align with the samples’ total bioactive compounds and antioxidant capacity described above. The antioxidant activity of the *M. bicolor* honey at 1 and 2 mg·mL^−1^ was comparable to that of ascorbic acid (the reference antioxidant). The *M. mondury* honey showed comparable antioxidant activity at 1 mg·mL^−1^ (from both seasons) and 2 mg·mL^−1^ (from the dry season). Still, it exhibited more substantial hemolysis prevention overall than the *M. bicolor* honey (Figure 2b). Notably, the *M. mondury* honey from the rainy season, at 2 mg·mL^−1^, displayed significantly higher antioxidant activity (*p* < 0.05) compared to that of all the other samples at different concentrations and ascorbic acid levels (Figure 2b,c).

Studies on the cellular protective effects of Brazilian stingless bee honey (SBH) are scarce. Alvarez-Suarez et al. (2012) demonstrated that phenolic-rich honey extracts significantly protected erythrocyte membranes from AAPH-induced hemolysis (50 mM) in a concentration- and time-dependent manner. This protective effect was likely due to the ability of phenolics to incorporate themselves into cell membranes or cross them to support cellular function [59]. More recently, honey from the Malaysian stingless bee *Trigona itama* was shown to have antioxidant, anti-inflammatory, and genoprotective effects. At concentrations from 0.2% to 0.8% (*v*/*v*), this honey protected WIL2-NS cells from H_2_O_2_-induced oxidative damage, and at 0.5% and 1%, it suppressed iNOS expression and NO production in RAW 264.7 cells [60]. Additionally, Manuka honey (New Zealand) has been shown to protect human dermal fibroblasts from oxidative damage and promote in vitro wound healing potential by enhancing the antioxidant response and mitochondrial function through AMPK/Nrf2 signaling activation [61].

Together, the findings described above and in the present study highlight the pharmacological potential of honey from various origins to modulate antioxidant mechanisms and offer health benefits. As is common with natural products, the seasonal and environmental conditions in the honey collection area might influence its composition and, consequently, its pharmacological properties. However, in the present study, we found that the antioxidant activity of the SBH was not substantially affected by seasonal changes based on the pluviometric index, suggesting the potential for the consistent bioactivity of SBH regardless of the collection period.

### 3.6. Discriminant Analysis of Stingless Bee Honey Samples

In the chemometric analysis, multivariate statistical methods were employed to differentiate the honey samples based on the seasonality, using data from the physicochemical properties, mineral profile, antioxidant capacity, and MS-based fingerprinting. The fingerprinting of the SBH samples is shown in the Supplementary Materials (Figures S1 and S2). Initially, the samples were categorized into four groups according to the collection period: spring, summer, autumn, and winter. Despite this classification, multivariate analysis indicated that the investigated variables failed to distinguish the samples between the four seasons, irrespective of the bee species analyzed (Figure S3a). However, upon closer examination of the data, a tendency for separation was observed, aligning with Brazil’s pluviometry patterns. With its extensive territory and rich biodiversity, Brazil occupies equatorial, tropical, and sub-tropical zones, leading to primarily warm-to-hot weather patterns [62]. In addition, the seasons are dominated by the movement of the tropical rain belt, resulting in dry and rainy seasons instead of the four-season pattern (spring, summer, autumn, and winter) observed in temperate zones [63]. In the present study, the climatic feature may have hindered the differentiation of the samples collected across the four seasons. Thus, a new approach was applied using a pluviometric-based seasonal classification (the rainy or dry season).

**Figure 3:**
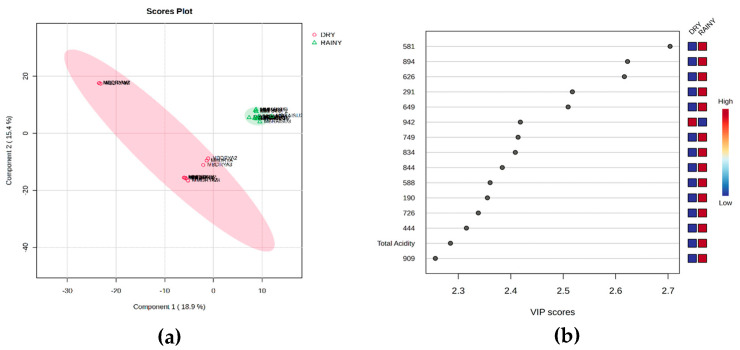
Discriminant analysis of stingless bee honey (SBH) based on seasonality, comparing the dry season (FS) and rainy season (QU). PLS-DA score plots (**a**) and VIP score plots (**b**) highlight the contributions of the physicochemical parameters, mineral profile, antioxidant capacity, and honey MS-based fingerprinting analysis.

A low-level data fusion strategy was used to construct the dataset of independent variables (X). Data from multiple analyses were processed separately through normalization, concatenated, and compiled into an independent data matrix. The resulting low-level data matrix was then autoscaled for further analysis. The dataset comprised 24 honey samples, including 8 samples (4 from each season—rainy and dry) for each stingless bee species (*M. mondury* and *M. bicolor*), tested in triplicate. These samples were grouped into two categories: the dry season (autumn and winter) and rainy season (spring and summer), with 229 variables representing chemical, physicochemical, and antioxidant assessments. PCA served as the initial exploratory data analysis method. Results from the PCA were inconclusive due to the significant overlap among most of the samples (Figure S3b), revealing the need for a more refined discriminant approach to achieve more evident differentiation. The data were subsequently transformed into triplicate means and analyzed using the PLS-DA supervised method (Figure 3). This approach enabled the simultaneous evaluation of all the investigated aspects of the honey samples, identifying essential analytical methods for characterizing SBH and the key factors for distinguishing samples between groups [7].

In the PLS-DA model (Figure 3a), the explained variance was 34.3% (two factors), with an R^2^ of 1.0, a Q^2^ of 0.8, and an accuracy of 0.9. These metrics indicate the model’s robustness and lack of overfitting, effectively separating the samples into dry- and rainy-season groups. Key contributors to this separation were the *m*/*z* 581, 894, 626, 291, and 649 ions, all with VIP scores exceeding 2.5 (Figure 3b), emphasizing their importance in group differentiation. Notably, except for the total acidity and water content, other physicochemical parameters were not significant variables in the group separation within the pluviometric-based chemometric approach. In a previous study, the iron content, rather than *m*/*z* ions, was identified as the primary factor distinguishing SBH from different biogeographical zones in Brazil. In that context, the mineral composition of the honey reflected the soil where the nectar-producing plants grew [7,54]. This study collected samples from the same location at different times, making the soil composition less likely to vary. These findings suggest that mineral content analysis could serve as a useful tool for identifying the geographic origin of honey, while fingerprinting secondary metabolites shows promise for determining the period of honey collection.

## 4. Conclusions

Based on the findings described here, the physicochemical and antioxidant characteristics of stingless bee honey (SBH) were influenced by seasonal variations, emphasizing the impact of environmental factors on the honey composition. Honey samples collected during the rainy and dry seasons displayed distinct differences in their moisture contents, total soluble solids, and acidities, which correlated with seasonal climatic conditions. The antioxidant activity was consistent regardless of the season, underlining the robust bioactivity of SBH. MS-based fingerprinting emerged as a promising method for determining the period of honey collection. These results suggest that chemometric approaches integrating seasonal and compositional data provide valuable tools for distinguishing honey characteristics and their environmental influences, advancing the understanding and potential applications of SBH in authenticity, nutrition, and health contexts. These findings may contribute to the development of identity and quality standards for stingless bee honey, highlighting its physicochemical and bioactive characteristics.

## Figures and Tables

**Table 1:** *Melipona* species and honey harvest periods.

Stingless Bee	Popular Name	Biogeographical Zone	Period of Harvest (Season)
*M. mondury*	Uruçú-Amarela	Atlantic Forest	January 2019 (Rainy)
			December 2019 (Rainy)
			April 2018 (Dry)
			July 2018 (Dry)
*M. bicolor*	Pé-de-Pau	Atlantic Forest	December 2019 (Rainy)
			March 2021 (Rainy)
			April 2018 (Dry)
			July 2018 (Dry)

**Table 2:** Physicochemical parameters of *M. mondury* and *M. bicolor* honeys collected during the dry and rainy seasons of the Atlantic Forest region.

SB	Season	MC (%)	Aw	TSS (°Brix)	pH	Total Acidity (mEq·kg^−1^)	Free Acidity (mEq·kg^−1^)	BP (AU)	HMF (mg·Kg^−1^)	Pfund (mm)	Color
*M.* *mondury*	Rainy	35.76 ± 10.23 ^A,a^	0.80 ± 0.07 ^A,a^	61.50 ± 10.87 ^A,a^	3.15 ± 0.13 ^A,a^	247.35 ± 21.36 ^A,a^	91.57 ± 10.27 ^A,a^	0.13 ± 0.00 ^A,a^	5.35 ± 6.06 ^A,a^	13.50 ± 14.82 ^A,a^	Extra white
	Dry	27.25 ± 0.60 ^A,a^	0.72 ± 0.01 ^B,a^	70.99 ± 0.60 ^B,a^	3.50 ± 0.04 ^B,a^	89.18 ± 27.99 ^B,a^	46.01 ± 0.93 ^B,a^	0.13 ± 0.03 ^A,a^	1.92 ± 2.28 ^A,a^	9.21 ± 5.97 ^A,a^	Extra white
*M. bicolor*	Rainy	32.74 ± 0.26 ^A,a^	0.79 ± 0.02 ^A,a^	64.88 ± 0.21 ^A,a^	3.36 ± 0.03 ^A,a^	270.69 ± 6.90 ^A,a^	98.07 ± 2.77 ^A,a^	0.14 ± 0.01 ^A,a^	4.26 ± 4.6 ^A,a^	8.32 ± 9.13 ^A,a^	Extra white
	Dry	31.56 ± 1.68 ^A,b^	0.75 ± 0.01 ^A,b^	66.43 ± 1.66 ^A,b^	3.49 ± 0.20 ^A,a^	195.20 ± 42.39 ^B,a^	106.73 ± 0.13 ^A,b^	0.17 ± 0.05 ^A,a^	1.16 ± 1.49 ^A,a^	37.34 ± 16.32 ^B,b^	Extra light amber

Values are expressed as mean ± standard deviation. To verify differences between means, ANOVA and Tukey’s test (*p* < 0.05) (parametric data) or the Kruskal–Wallis test (non-parametric data) were performed, followed by Dunn’s post hoc test (*p* < 0.05). ^A,B^ Different capital letters superscripted in the same column show significant differences between the same species in different seasons (i.e., compare the same species in different seasons). ^a,b^ Different superscript lowercase letters in the same column show significant differences between the different species in the same season (i.e., compare different species in the same season). SB, stingless bee; MC, moisture; Aw, water activity; TSS, total soluble solids; BP, brown pigments; HMF, hydroxymethylfurfural; AU, arbitrary unit.

## Data Availability

The original contributions presented in this study are included in the article/Supplementary Materials. Further inquiries can be directed to the corresponding authors.

## Supplementary Materials

The following supporting information can be downloaded at https://www.mdpi.com/article/10.3390/foods14020305/s1, Table S1: Correlation matrix between physicochemical, phenolic, and antioxidant capacities of *M. mondury* and *M. bicolor* honeys; Figure S1: Mass spectra of stingless bee honey from *M. mondury* collected during the rainy season; Figure S2: Mass spectra of stingless bee honey from *M. bicolor* collected during the rainy season; Figure S3: Discriminant analysis of stingless bee honey (SBH) based on seasonality using unsupervised Principal Component Analysis (PCA).

## References

[1] Macedo C.R.d.C., Aquino I.d.S., Borges P.d.F., Barbosa A.d.S., de Medeiros G.R. (2021). Nesting Behavior of Stingless Bees. Ciênc. Anim. Bras..

[2] Jaffé R., Pope N., Carvalho A.T., Maia U.M., Blochtein B., de Carvalho C.A.L., Carvalho-Zilse G.A., Freitas B.M., Menezes C., Ribeiro M.d.F. (2015). Bees for Development: Brazilian Survey Reveals How to Optimize Stingless Beekeeping. PLoS ONE.

[3] de Sousa J.M.B., de Souza E.L., Marques G., de Toledo Benassi M., Gullón B., Pintado M.M., Magnani M. (2016). Sugar Profile, Physicochemical and Sensory Aspects of Monofloral Honeys Produced by Different Stingless Bee Species in Brazilian Semi-Arid Region. LWT.

[4] da Silva I.A.A., da Silva T.M.S., Camara C.A., Queiroz N., Magnani M., de Novais J.S., Soledade L.E.B., Lima E.d.O., de Souza A.L., de Souza A.G. (2013). Phenolic Profile, Antioxidant Activity and Palynological Analysis of Stingless Bee Honey from Amazonas, Northern Brazil. Food Chem..

[5] Zapata-Hernández G., Gajardo-Rojas M., Calderón-Seguel M., Muñoz A.A., Yáñez K.P., Requier F., Fontúrbel F.E., Ormeño-Arriagada P.I., Arrieta H. (2024). Advances and Knowledge Gaps on Climate Change Impacts on Honey Bees and Beekeeping: A Systematic Review. Glob. Change Biol..

[6] Tennakoon S., Apan A., Maraseni T. (2024). Unravelling the Impact of Climate Change on Honey Bees: An Ensemble Modelling Approach to Predict Shifts in Habitat Suitability in Queensland, Australia. Ecol. Evol..

[7] Lavinas F.C., Gomes B.A., Silva M.V., Nunes R.M., Leitão S.G., Moura M.R., Simas R.C., Carneiro C.S., Rodrigues I.A. (2023). Discriminant Analysis of Brazilian Stingless Bee Honey Reveals an Iron-Based Biogeographical Origin. Foods.

[8] Ávila S., Hornung P.S., Teixeira G.L., Malunga L.N., Apea-Bah F.B., Beux M.R., Beta T., Ribani R.H. (2019). Bioactive Compounds and Biological Properties of Brazilian Stingless Bee Honey Have a Strong Relationship with the Pollen Floral Origin. Food Res. Int..

[9] Biluca F.C., Braghini F., Gonzaga L.V., Costa A.C.O., Fett R. (2016). Physicochemical Profiles, Minerals and Bioactive Compounds of Stingless Bee Honey (*Meliponinae*). J. Food Compos. Anal..

[10] do Nascimento A.S., Marchini L.C., de Carvalho C.A.L., Araújo D.F.D., de Olinda R.A., da Silveira T.A. (2015). Physical-Chemical Parameters of Honey of Stingless Bee (Hymenoptera: Apidae). Chem. Sci. Int. J..

[11] Sant’Ana R.d.S., de Carvalho C.A.L., Oda-Souza M., Souza B.d.A., Dias F.d.S. (2020). Characterization of Honey of Stingless Bees from the Brazilian Semi-Arid Region. Food Chem..

[12] Zulkhairi Amin F.A., Sabri S., Mohammad S.M., Ismail M., Chan K.W., Ismail N., Norhaizan M.E., Zawawi N. (2018). Therapeutic Properties of Stingless Bee Honey in Comparison with European Bee Honey. Adv. Pharmacol. Pharm. Sci..

[13] Ávila S., Beux M.R., Ribani R.H., Zambiazi R.C. (2018). Stingless Bee Honey: Quality Parameters, Bioactive Compounds, Health-Promotion Properties and Modification Detection Strategies. Trends Food Sci. Technol..

[14] Mahmood A.L., Lani M.N., Hassan Z., Razak S.B.A., Ahmad F.T. (2021). Antioxidant and Antimicrobial Properties of Indo-Malayan Stingless Bee (*Heterotrigona itama*) Honey from Different Seasons and Distribution of Flowers. Food Res..

[15] Suhri A.G.M.I., Bahar I. (2023). Water Content of Stingless Bee Honey Varies by Season. J. Biol. Trop..

[16] Wu M.-C., Wu C.-Y., Klaithin K., Tiong K.K., Peng C.-C. (2022). Effect of Harvest Time Span on Physicochemical Properties, Antioxidant, Antimicrobial, and Anti-Inflammatory Activities of Meliponinae Honey. J. Sci. Food Agric..

[17] Zielinski A.A.F., Haminiuk C.W.I., Nunes C.A., Schnitzler E., van Ruth S.M., Granato D. (2014). Chemical Composition, Sensory Properties, Provenance, and Bioactivity of Fruit Juices as Assessed by Chemometrics: A Critical Review and Guideline. Compr. Rev. Food Sci. Food Saf..

[18] AOAC—Association of Official Analytical Chemists (2012). Official Methods of Analysis of AOAC International.

[19] Ferreira I.C.F.R., Aires E., Barreira J.C.M., Estevinho L.M. (2009). Antioxidant Activity of Portuguese Honey Samples: Different Contributions of the Entire Honey and Phenolic Extract. Food Chem..

[20] Turkmen N., Sari F., Poyrazoglu E.S., Velioglu Y.S. (2006). Effects of Prolonged Heating on Antioxidant Activity and Colour of Honey. Food Chem..

[21] Klockenkämper R., von Bohlen A. (1996). Elemental Analysis of Environmental Samples by Total Reflection X-Ray Fluorescence: A Review. X-Ray Spectrom..

[22] Meda A., Lamien C.E., Romito M., Millogo J., Nacoulma O.G. (2005). Determination of the Total Phenolic, Flavonoid and Proline Contents in Burkina Fasan Honey, as Well as Their Radical Scavenging Activity. Food Chem..

[23] Benzie I.F.F., Strain J.J. (1996). The Ferric Reducing Ability of Plasma (FRAP) as a Measure of “Antioxidant Power”: The FRAP Assay. Anal. Biochem..

[24] Park S.K., Lee Y.K. (2021). Antioxidant Activity in Rheum Emodi Wall (Himalayan Rhubarb). Molecules.

[25] Lima R., Silva M.V.T., Gomes B.A., Macedo E.H.B.C., Santana M.N., Amaral A.C.F., Silva J.R.A., Corrêa P.G., Godoy R.L.O., Santiago M.C.P.A. (2023). Chemical Profile and Hematoprotective Activity of Artisanal Jabuticaba (*Plinia jabuticaba*) Wine and Derived Extracts. Fermentation.

[26] Sharin S.N., Abdullah Sani M.S., Kassim N.K., Yuswan M.H., Abd Aziz A., Jaafar M.A., Hashim A.M. (2024). Impact of Harvesting Seasons on Physicochemical Properties and Volatile Compound Profiles of Malaysian Stingless Bee Honey Analysed Using Chemometrics and Support Vector Machine. Food Chem..

[27] Marcolin L.C., Lima L.R., de Oliveira Arias J.L., Berrio A.C.B., Kupski L., Barbosa S.C., Primel E.G. (2021). Meliponinae and *Apis Mellifera* Honey in Southern Brazil: Physicochemical Characterization and Determination of Pesticides. Food Chem..

[28] FAO Food and Agriculture Organization of the United Nations. Standards|CODEXALIMENTARIUS FAO-WHO. https://www.fao.org/fao-who-codexalimentarius/codex-texts/list-standards/en/.

[29] Brazil. Ministério da Agricultura e Pecuária Instrução Normativa Mapa n° 11, de 20 de Outubro de 2000—Regulamento Técnico de Identidade e Qualidade do Mel. https://www.gov.br/agricultura/pt-br/assuntos/defesa-agropecuaria/suasa/regulamentos-tecnicos-de-identidade-e-qualidade-de-produtos-de-origem-animal-1/rtiq-mel-e-produtos-apicolas.

[30] Gomes V.V., Bandeira A.M.P., Cordovil K.P.S., Filho J.d.R.B., Braghini F., Biluca F.C., Gonzaga L.V., Fett R., da Costa K.S., de Azevedo M.M.R. (2022). Physicochemical Characterization and Antioxidant Activity of Honey Samples of *Apis Mellifera* and Different Species of Meliponinae Subfamily from the Brazilian Eastern Amazon Region. Food Sci. Technol..

[31] Nordin A., Sainik N.Q.A.V., Chowdhury S.R., Saim A.B., Idrus R.B.H. (2018). Physicochemical Properties of Stingless Bee Honey from around the Globe: A Comprehensive Review. J. Food Compos. Anal..

[32] Evangelista-Rodrigues A., da Silva E.M.S., Beserra E.M.F., Rodrigues M.L. (2005). Análise físico-química dos méis das abelhas Apis mellifera e Melipona scutellaris produzidos em regiões distintas no Estado da Paraíba. Cienc. Rural.

[33] Souza B.d.A., de Carvalho C.A.L., Sodré G.d.S., Marchini L.C. (2004). Características físico-químicas de amostras de mel de Melipona asilvai (Hymenoptera: Apidae). Cienc. Rural.

[34] dos Santos A.C., Biluca F.C., Braghini F., Gonzaga L.V., Costa A.C.O., Fett R. (2021). Phenolic Composition and Biological Activities of Stingless Bee Honey: An Overview Based on Its Aglycone and Glycoside Compounds. Food Res. Int..

[35] Vit P., Pedro S.R.M., Roubik D. (2013). Pot-Honey: A Legacy of Stingless Bees.

[36] Hassan K.N.A.M., Ibrahim R.K.R., Maisarah D., Zakaria Z., Ihsan N., Fauziah T.A. (2021). Profiling pH and Moisture Content of Stingless Bee Honey in Closed and Opened Cerumen Honey Pots. J. Phys. Conf. Ser..

[37] Carvalho C.A.L., Sodré G.S., Fonseca A.A.O., Alves R.M.O., Souza B.A., Clarton L. (2009). Physicochemical Characteristics and Sensory Profile of Honey Samples from Stingless Bees (Apidae: Meliponinae) Submitted to a Dehumidification Process. An. Acad. Bras. Ciênc..

[38] Gela A., Hora Z., Kebebe D., Gebresilassie A. (2021). Physico-chemical characteristics of honey produced by stingless bees (Meliponula beccarii) from West Showa zone of Oromia Region, Ethiopia. Heliyon.

[39] Chuttong B., Chanbang Y., Sringarm K., Burgett M. (2016). Physicochemical Profiles of Stingless Bee (Apidae: Meliponini) Honey from South East Asia (Thailand). Food Chem..

[40] Vieira T.R., Noguez C.S., dos Santos M.A., Wagner S.A. (2023). Caracterização físico-química e botânica do mel de abelhas sem ferrão (Meliponini), de ocorrência no Vale do Taquari—RS, objetivando edição de RTIQ. Res. Soc. Dev..

[41] Guerrini A., Bruni R., Maietti S., Poli F., Rossi D., Paganetto G., Muzzoli M., Scalvenzi L., Sacchetti G. (2009). Ecuadorian Stingless Bee (Meliponinae) Honey: A Chemical and Functional Profile of an Ancient Health Product. Food Chem..

[42] Biluca F.C., Della Betta F., de Oliveira G.P., Pereira L.M., Gonzaga L.V., Costa A.C.O., Fett R. (2014). 5-HMF and Carbohydrates Content in Stingless Bee Honey by CE before and after Thermal Treatment. Food Chem..

[43] Pasias I.N., Kiriakou I.K., Proestos C. (2017). HMF and Diastase Activity in Honeys: A Fully Validated Approach and a Chemometric Analysis for Identification of Honey Freshness and Adulteration. Food Chem..

[44] Pita-Calvo C., Guerra-Rodríguez M.E., Vázquez M. (2017). Analytical Methods Used in the Quality Control of Honey. J. Agric. Food Chem..

[45] Terrab A., Recamales A.F., Hernanz D., Heredia F.J. (2004). Characterisation of Spanish Thyme Honeys by Their Physicochemical Characteristics and Mineral Contents. Food Chem..

[46] Zhou X., Taylor M.P., Salouros H., Prasad S. (2018). Authenticity and Geographic Origin of Global Honeys Determined Using Carbon Isotope Ratios and Trace Elements. Sci. Rep..

[47] Czipa N., Andrási D., Kovács B. (2015). Determination of Essential and Toxic Elements in Hungarian Honeys. Food Chem..

[48] Folkeson L., Nyholm N.E.I., Tyler G. (1990). Influence of Acidity and Other Soil Properties on Metal Concentrations in Forest Plants and Animals. Sci. Total Environ..

[49] Squadrone S., Brizio P., Stella C., Mantia M., Pederiva S., Brusa F., Mogliotti P., Garrone A., Abete M.C. (2020). Trace Elements and Rare Earth Elements in Honeys from the Balkans, Kazakhstan, Italy, South America, and Tanzania. Env. Sci. Pollut. Res..

[50] Batista B.L., da Silva L.R.S., Rocha B.A., Rodrigues J.L., Berretta-Silva A.A., Bonates T.O., Gomes V.S.D., Barbosa R.M., Barbosa F. (2012). Multi-Element Determination in Brazilian Honey Samples by Inductively Coupled Plasma Mass Spectrometry and Estimation of Geographic Origin with Data Mining Techniques. Food Res. Int..

[51] NIH National Institutes of Health, Office of Dietary Supplements. Nutrient Recommendations: Dietary Reference Intakes (DRI). U.S. Department of Health & Human Services 2019. https://ods.od.nih.gov/HealthInformation/nutrientrecommendations.aspx.

[52] AHA American Heart Association. Added Sugars. https://www.heart.org/en/healthy-living/healthy-eating/eat-smart/sugar/added-sugars.

[53] Brazil. Agência Nacional de Vigilância Sanitária (ANVISA). Instrução Normativa N° 75, de 8 de Outubro de 2020. https://anvisalegis.datalegis.net/action/ActionDatalegis.php?acao=categorias&cod_modulo=310&menuOpen=true.

[54] Pucholobek G., de Andrade C.K., Rigobello E.S., Wielewski P., de Toledo V.d.A.A., Quináia S.P. (2022). Determination of the Ca, Mn, Mg and Fe in Honey from Multiple Species of Stingless Bee Produced in Brazil. Food Chem..

[55] Umami N., Agus A. (2022). Stingless Bee Honey (*Tetragonula laeviceps*): Chemical Composition and Their Potential Roles as an Immunomodulator in Malnourished Rats. Saudi J. Biol. Sci..

[56] Shakoori Z., Salaseh E., Mehrabian A.R., Tehrani D.M., Dardashti N.F., Salmanpour F. (2024). The Amount of Antioxidants in Honey Has a Strong Relationship with the Plants Selected by Honey Bees. Sci. Rep..

[57] Moniruzzaman M., Sulaiman S.A., Khalil M.I., Gan S.H. (2013). Evaluation of Physicochemical and Antioxidant Properties of Sourwood and Other Malaysian Honeys: A Comparison with Manuka Honey. Chem. Cent. J..

[58] Arul Mozhi P., Doss V.A. (2023). Synergistic Antioxidant Effect of Cinnamomum Verum and Stingless Bee Honey. Biointerface Res. Appl. Chem..

[59] Alvarez-Suarez J.M., Giampieri F., González-Paramás A.M., Damiani E., Astolfi P., Martinez-Sanchez G., Bompadre S., Quiles J.L., Santos-Buelga C., Battino M. (2012). Phenolics from Monofloral Honeys Protect Human Erythrocyte Membranes against Oxidative Damage. Food Chem. Toxicol..

[60] Ooi T.C., Yaacob M., Rajab N.F., Shahar S., Sharif R. (2021). The Stingless Bee Honey Protects against Hydrogen Peroxide-Induced Oxidative Damage and Lipopolysaccharide-Induced Inflammation in Vitro. Saudi J. Biol. Sci..

[61] Alvarez-Suarez J.M., Giampieri F., Cordero M., Gasparrini M., Forbes-Hernández T.Y., Mazzoni L., Afrin S., Beltrán-Ayala P., González-Paramás A.M., Santos-Buelga C. (2016). Activation of AMPK/Nrf2 Signalling by Manuka Honey Protects Human Dermal Fibroblasts against Oxidative Damage by Improving Antioxidant Response and Mitochondrial Function Promoting Wound Healing. J. Funct. Foods.

[62] CCKP Climate Change Knowledge Portal. World Bank Climate Change Knowledge Portal. https://climateknowledgeportal.worldbank.org/.

[63] Hidalgo L.G. (1999). Analysis of the Fundamental Tropical Seasons by Combining Satellite and Raingauge Data. Meteorol. Appl..

