# Supplementary material for: Rainy and Dry Seasons Are Relevant Factors Affecting Chemical and Antioxidant Properties of Meliponini Honey
Source: Foods. 2025 Jan 17;14(2):305. doi: 10.3390/foods14020305 (PMC11765282; doi:10.3390/foods14020305)
Supplement: Supplementary file 1 [file foods-14-00305-s001.zip › foods-3366414-supplementary.pdf]

**Table S1.** Correlation matrix between physicochemical, phenolics, and antioxidant capacity of *M. mondury* and *M. bicolor* honeys.

| | **Aw** | **TSS** | **MC** | **DP** | **pH** | **FA** | **TA** | **HMF** | **Color** | **TPC** | **TFC** | **FRAP** | **ABTS** |
|---|---|---|---|---|---|---|---|---|---|---|---|---|---|
| **Aw** | **1** | | | | | | | | | | | | |
| **TSS** | **-0,903** | **1** | | | | | | | | | | | |
| **MC** | **0,846** | **-0,954** | **1** | | | | | | | | | | |
| **DP** | -0,045 | 0,071 | -0,143 | **1** | | | | | | | | | |
| **pH** | **-0,548** | **0,457** | **-0,403** | 0,347 | **1** | | | | | | | | |
| **FA** | **0,800** | **-0,839** | **0,861** | -0,202 | **-0,567** | **1** | | | | | | | |
| **TA** | **0,711** | **-0,570** | **0,454** | 0,040 | -0,331 | **0,535** | **1** | | | | | | |
| **HMF** | -0,274 | 0,268 | -0,291 | -0,022 | -0,242 | 0,051 | 0,041 | **1** | | | | | |
| **Color** | -0,143 | **0,420** | **-0,418** | 0,377 | 0,220 | -0,164 | -0,178 | -0,023 | **1** | | | | |
| **TPC** | **-0,431** | 0,059 | 0,052 | **-0,507** | 0,250 | 0,142 | 0,048 | 0,066 | 0,052 | **1** | | | |
| **TFC** | -0,007 | 0,158 | -0,236 | **0,589** | **0,432** | -0,163 | 0,398 | -0,036 | **0,481** | -0,012 | **1** | | |
| **FRAP** | 0,301 | -0,293 | 0,314 | 0,003 | 0,221 | **0,407** | **0,557** | 0,035 | 0,235 | **0,523** | **0,503** | **1** | |
| **ABTS** | 0,165 | -0,104 | 0,090 | 0,173 | 0,401 | 0,164 | **0,473** | -0,100 | 0,213 | 0,360 | **0,644** | **0,770** | **1** |

Aw = water activity; TSS = total soluble solids; MC = moisture content; DP = dark pigments; FA = free acidity; TA = total acidity; HMF = hydroxymethylfurfural; TPC = total phenolic content; TF = total flavonoids. The data are expressed as the correlation coefficient, r. An r value close to +1 or -1 indicates a strong relationship, while a value near 0 indicates a weak or no relationship. Correlations in bold are significant ($p < 0.05$).

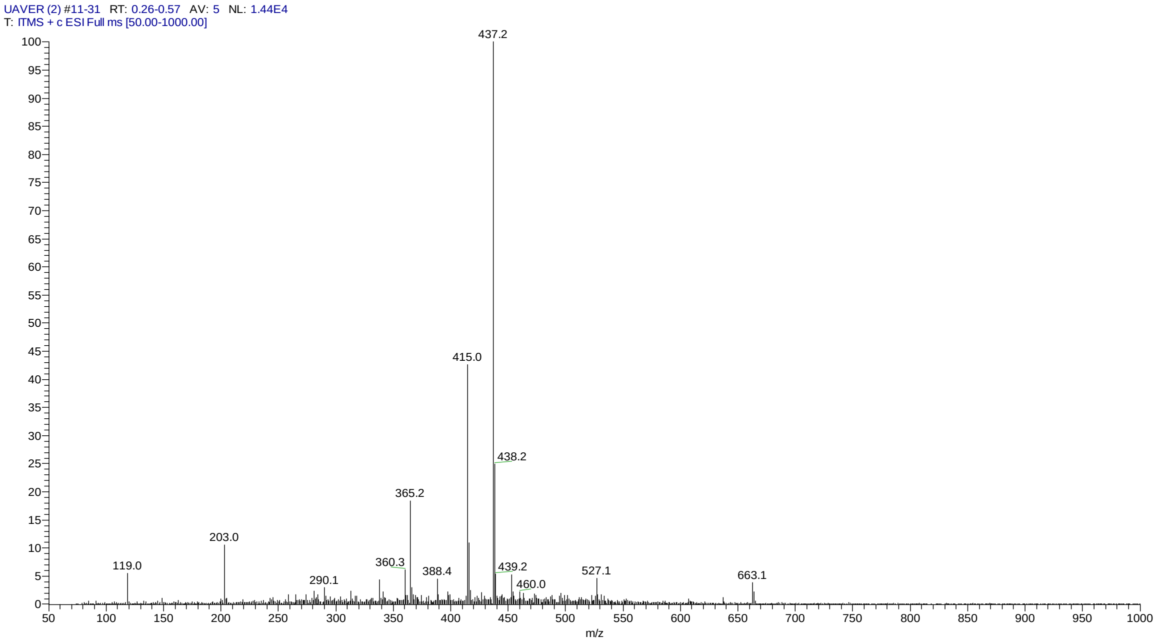


**(a)**

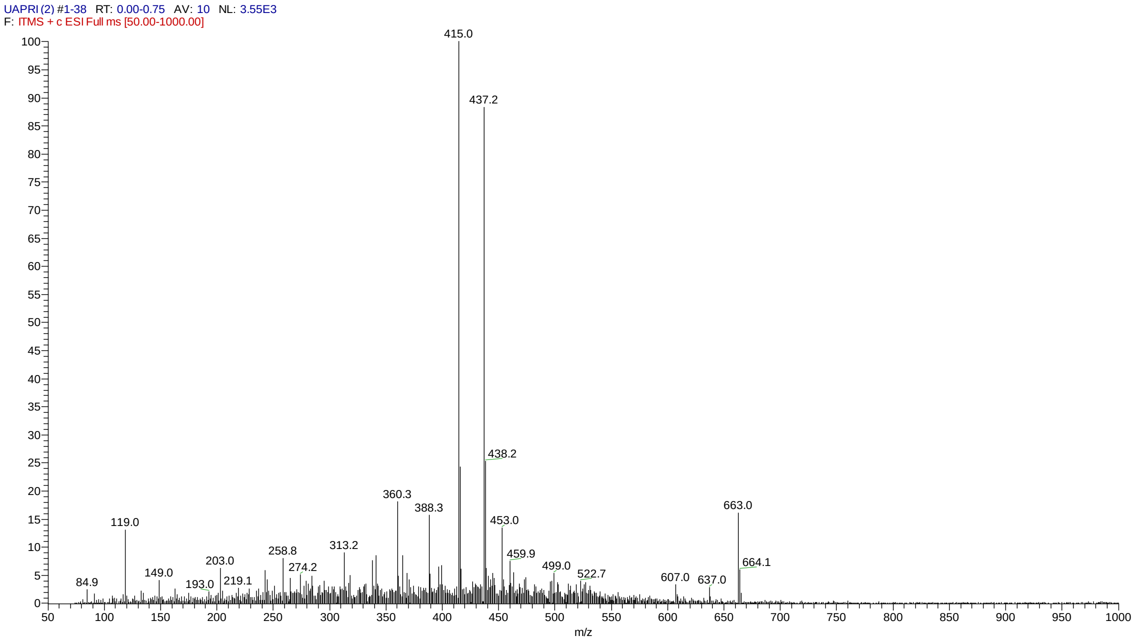


**(b)**

**Figure S1.** Mass spectra of stingless bee honey from *Melipona mondury* collected during the rainy season. (a) summer sample; (b) spring sample.

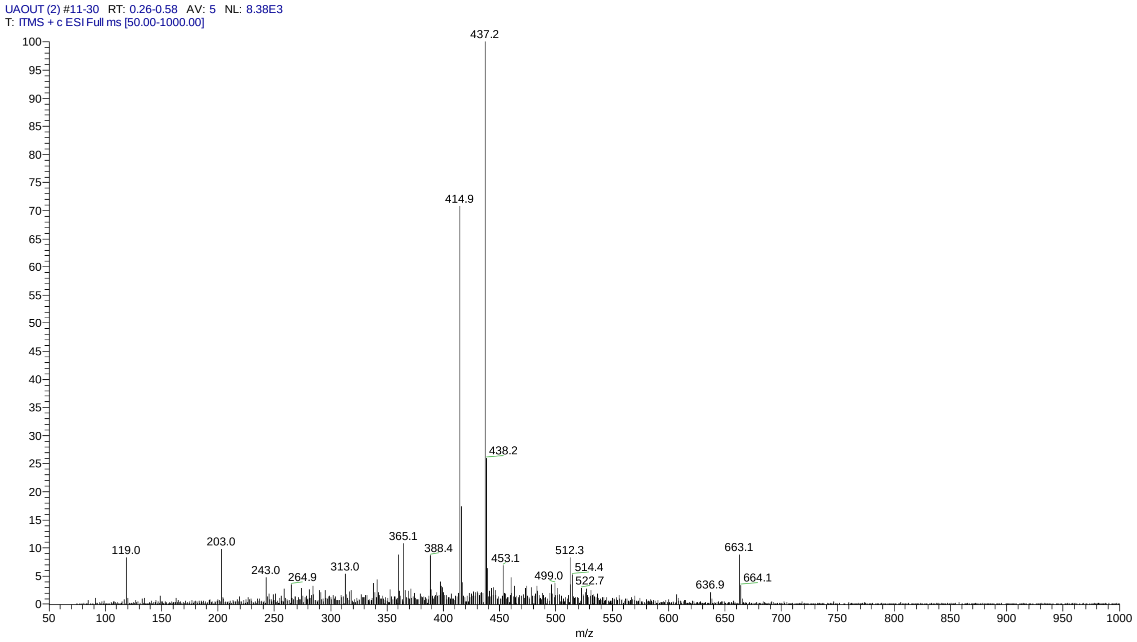


**(c)**

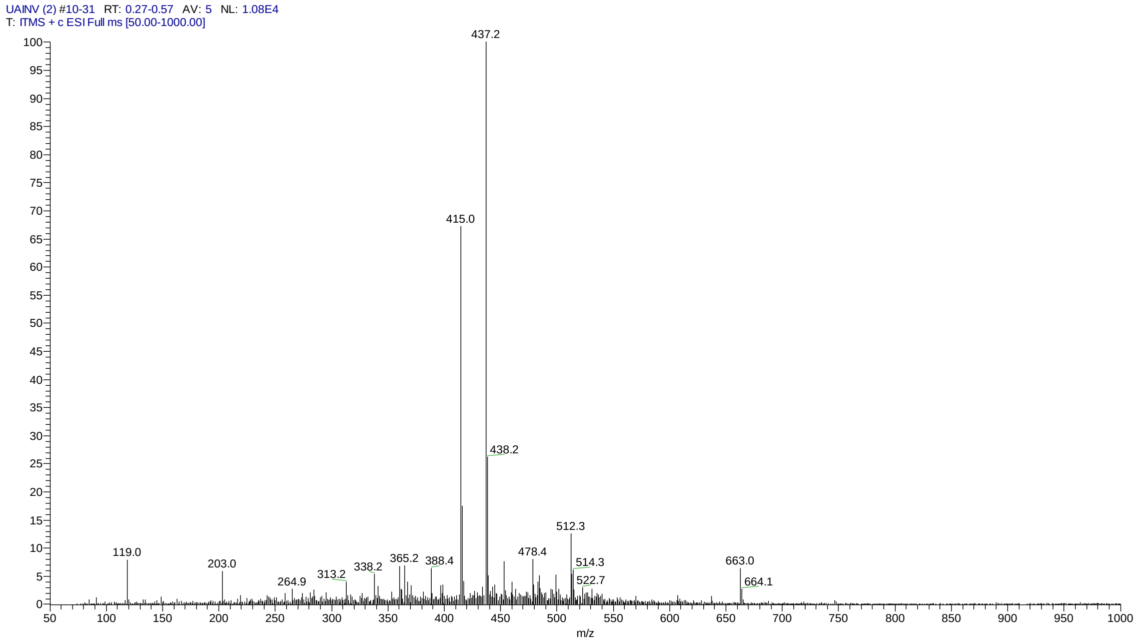


**(d)**

**Figure S1 (continued).** Mass spectra of stingless bee honey from *Melipona mondury* collected during the dry season. (c) autumn sample; (d) winter sample.

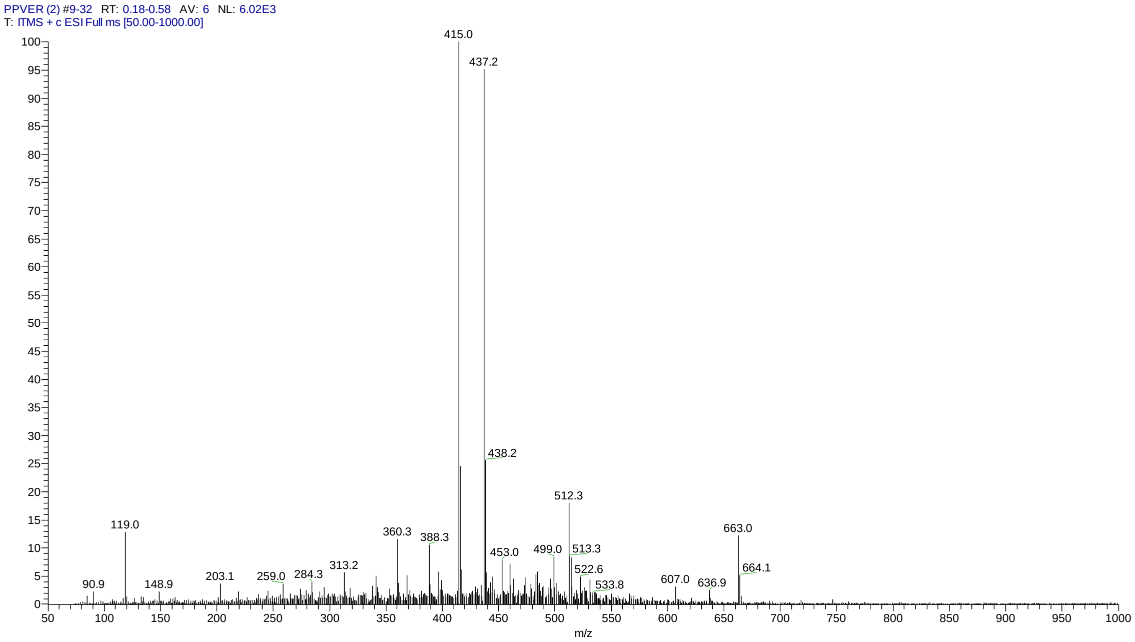


**(a)**

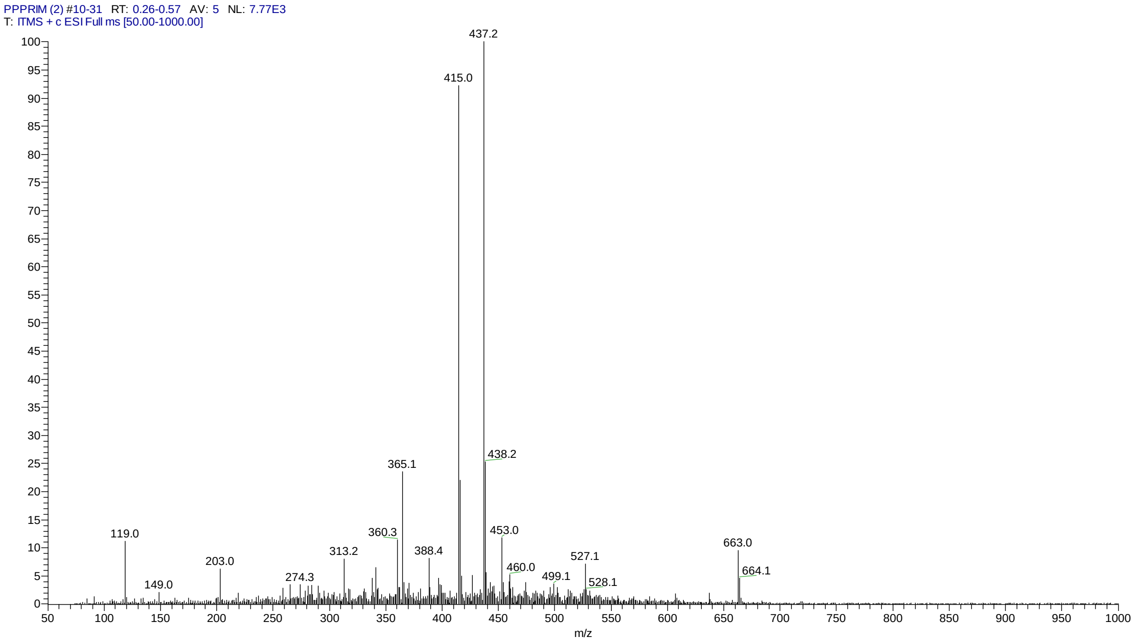


**(b)**

**Figure S2.** Mass spectra of stingless bee honey from *Melipona bicolor* collected during the rainy season. (a) summer sample; (b) spring sample.

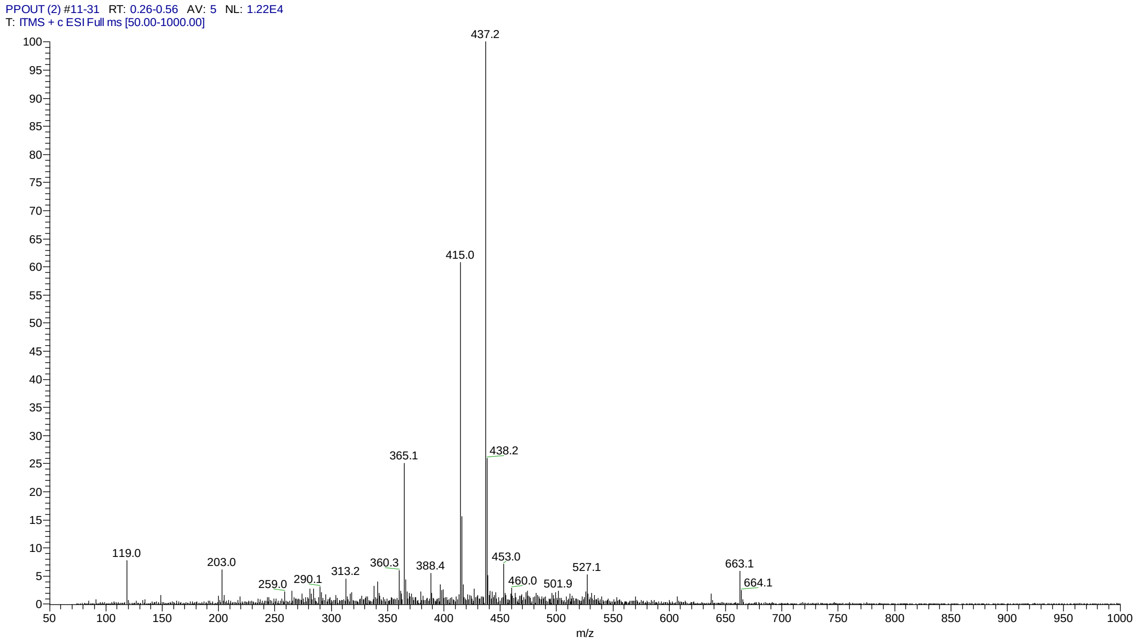


**(c)**

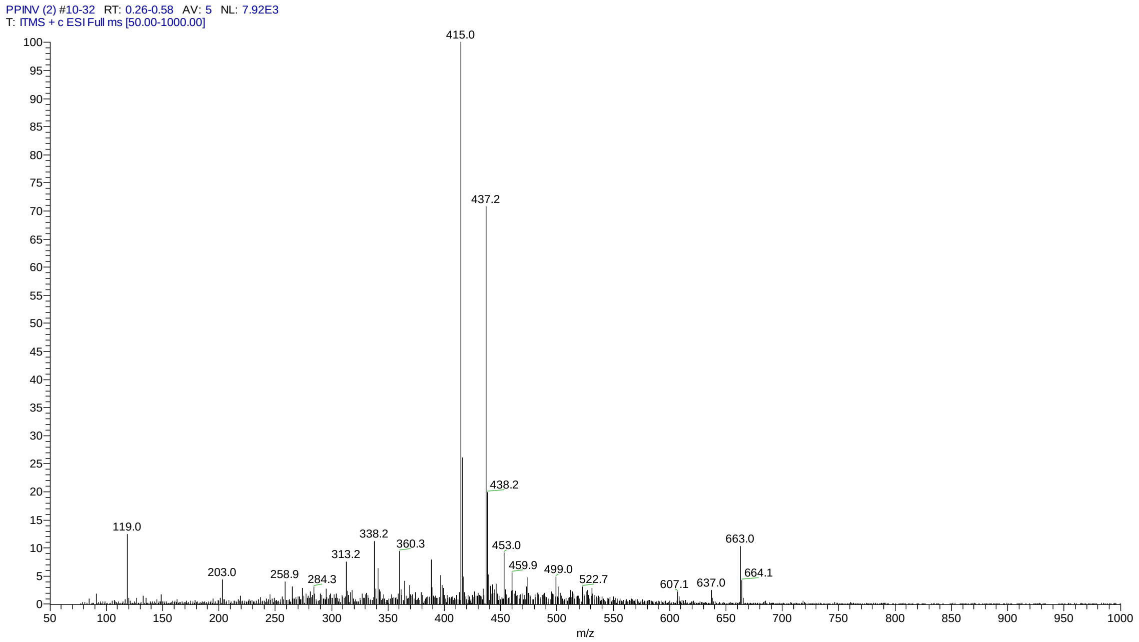


**(d)**

**Figure S2 (continued).** Mass spectra of stingless bee honey from *Melipona bicolor* collected during the dry season. (c) autumn sample; (d) winter sample.

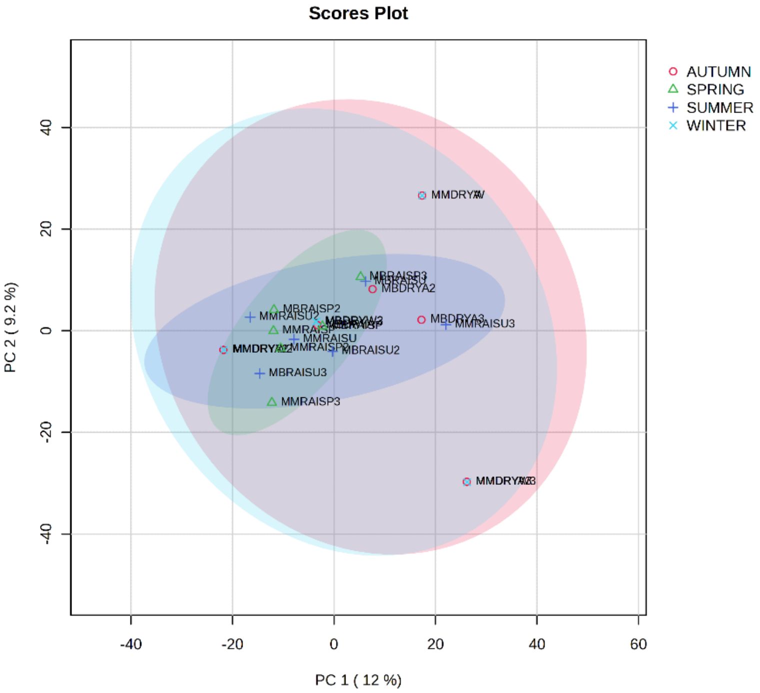


**(a)**

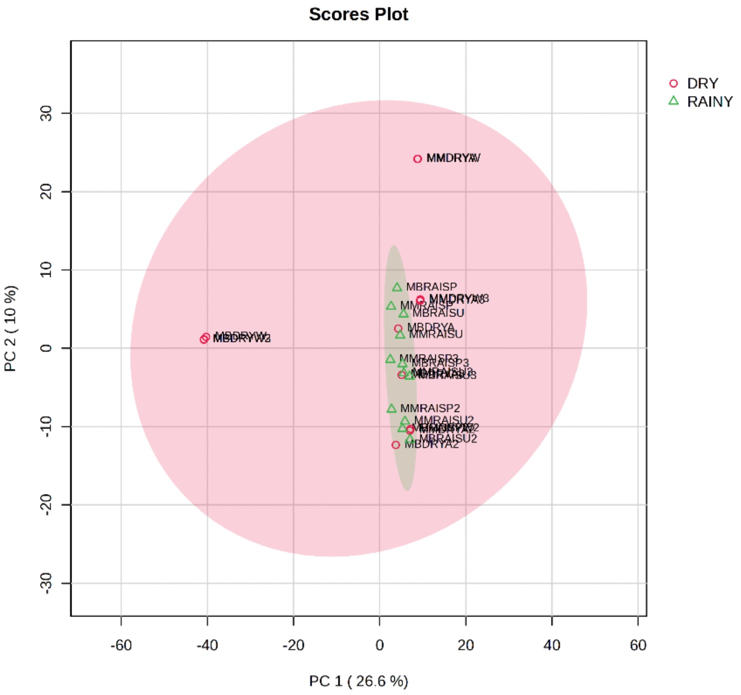


**(b)**

**Figure S3.** Discriminant analysis of stingless bee honey (SBH) based on seasonality using unsupervised Principal Component Analysis (PCA). (a) PCA applied to the traditional four-season classification (spring, summer, autumn, and winter); (b) PCA applied to the pluviometric-based seasonal classification (rainy and dry seasons). The overlap of ellipses indicates that the unsupervised analysis could not effectively distinguish between the honey samples.
